# Immune and Neuroprotective Effects of Physical Activity on the Brain in Depression

**DOI:** 10.3389/fnins.2018.00498

**Published:** 2018-07-26

**Authors:** Cristy Phillips, Atoossa Fahimi

**Affiliations:** ^1^Physical Therapy, Arkansas State University, Jonesboro, AR, United States; ^2^Physical Therapy, University of Tennessee Health Science Center, Memphis, TN, United States; ^3^Silverberry Genomics, San Francisco, CA, United States

**Keywords:** immune, stress, depression, physical activity, neuroprotection, peroxisome proliferator-activated receptor gamma coactivator 1-alpha, growth factors, glutamate

## Abstract

Physical activity—a lifestyle factor that is associated with immune function, neuroprotection, and energy metabolism—modulates the cellular and molecular processes in the brain that are vital for emotional and cognitive health, collective mechanisms that can go awry in depression. Physical activity optimizes the stress response, neurotransmitter level and function (e.g., serotonergic, noradrenergic, dopaminergic, and glutamatergic), myokine production (e.g., interleukin-6), transcription factor levels and correlates [e.g., peroxisome proliferator-activated receptor C coactivator-1α [PGC-1α], mitochondrial density, nitric oxide pathway activity, Ca^2+^ signaling, reactive oxygen specie production, and AMP-activated protein kinase [AMPK] activity], kynurenine metabolites, glucose regulation, astrocytic health, and growth factors (e.g., brain-derived neurotrophic factor). Dysregulation of these interrelated processes can effectuate depression, a chronic mental illness that affects millions of individuals worldwide. Although the biogenic amine model has provided some clinical utility in understanding chronic depression, a need remains to better understand the interrelated mechanisms that contribute to immune dysfunction and the means by which various therapeutics mitigate them. Fortunately, convergent evidence suggests that physical activity improves emotional and cognitive function in persons with depression, particularly in those with comorbid inflammation. Accordingly, the aims of this review are to (1) underscore the link between inflammatory correlates and depression, (2) explicate immuno-neuroendocrine foundations, (3) elucidate evidence of neurotransmitter and cytokine crosstalk in depressive pathobiology, (4) determine the immunomodulatory effects of physical activity in depression, (5) examine protocols used to effectuate the positive effects of physical activity in depression, and (6) highlight implications for clinicians and scientists. It is our contention that a deeper understanding of the mechanisms by which inflammation contributes to the pathobiology of depression will translate to novel and more effective treatments, particularly by identifying relevant patient populations that can benefit from immune-based therapies within the context of personalized medicine.

## Introduction

Depression is a pervasive health problem that includes emotional, psychomotor, cognitive, and biorhythmic disturbances (Kessler et al., 2005), symptoms that are associated with a 20-fold increase in the risk of suicide (Lépine and Briley, 2011). Current estimates suggest that close to 300 million persons are affected worldwide (Ferrari et al., 2013a), making depression the leading cause of disability as measured by disability-adjusted life years (Reddy, 2010). In addition to the incredible personal toll, the direct and indirect costs of treating depression are staggering. Spending on depression-related costs is $83.1 billion annually in the United States (Greenberg et al., 2003).

Initial progress toward understanding the pathobiology of depression was made following the serendipitous discovery that amine modulation effectuated disturbances in mood (Ghasemi et al., 2017), a finding that suggested that depression was caused by deficits in monoamine function. Accordingly, the majority of therapies for treating depression were derived to target the monoaminergic system. Notwithstanding, extant therapeutics exert a slow pace of action (3–5 weeks), have extensive side effects, and fail to provide full symptom relief in a significant proportion of persons treated (Paul and Skolnick, 2003; Trivedi, 2006). These limitations implicated other factors in depression and prompted stakeholders to diversify their search for mechanisms, biomarkers, and treatments.

Among the alternative mechanisms and therapeutics that have garnered increased attention are immune mechanisms that promote the body's natural response to protect against injury, infection, and emotional stress. The brain regulates central and peripheral immune processes via modulation of neurotransmitters (e.g., serotonergic, noradrenergic, dopaminergic, and glutamatergic), endocrine hormones, and cytokines (nonstructural proteins that are secreted by distinct cell populations and that exert variable effects at multiple levels of the central nervous system, e.g., neuroendocrine, autonomic, and behavioral) (Besedovsky and del Rey, 1992; Niciu et al., 2014; Ghasemi et al., 2017), processes that can go awry in the case of depression.

Indeed, a bevy of research indicates a link between inflammation and depression. Preclinical study demonstrates that stress paradigms (e.g., chronic unpredictable stress, learned helplessness, social defeat, and social isolation) induce pro-inflammatory cytokines centrally and peripherally (Steptoe et al., 2001; Bartolomucci et al., 2003; Grippo et al., 2005; Chourbaji et al., 2006; Audet et al., 2011; Gómez-Lázaro et al., 2011; Moller et al., 2013), changes that correlate with depressive-like symptoms (Kenis and Maes, 2002; Tuglu et al., 2003; Basterzi et al., 2005; Tsao et al., 2006; Dantzer et al., 2008; Miller et al., 2009; Elgarf et al., 2014; Lu et al., 2017) but can be mitigated following antidepressant administration (Dantzer et al., 2008; Guo et al., 2009; Miller et al., 2009; Elgarf et al., 2014; Lu et al., 2017). Others have shown that genetically modified rodents with impaired pro-inflammatory immune signaling fail to exhibit depressive-like behaviors that are induced in wild-type mice following chronic mild stress (Goshen et al., 2008; Brüning et al., 2015). Agents that induce inflammation (e.g., recombinant cytokines) in humans recapitulate symptoms of depression, effects that are circumvented with selective serotonin reuptake inhibitor (SSRI) administration (Hauser et al., 2002). In persons with autoimmune or inflammatory disorders, tumor necrosis factor (TNF) antagonists and certain nonsteroidal anti-inflammatory agents (Brunello et al., 2006; Müller et al., 2006; Tyring et al., 2006; Krishnan et al., 2007; Soczynska et al., 2009; Menter et al., 2010; Fond et al., 2014; Köhler et al., 2014; Abbott et al., 2015) exert antidepressant effects. In the general population, clinical evidence suggests an increased tendency for pro-inflammatory markers in persons with depression (increased interleukin [IL]-6, TNF-α, and CRP) relative to controls (Laske et al., 2008; Steiner et al., 2012), a trend that normalizes following response to antidepressants (Myint et al., 2005). Others have shown that administration of anti-inflammatory agents in combination with antidepressants accelerates and enhances treatment response in a subset of persons with depression (Mendlewicz et al., 2006; Müller et al., 2006; Nery et al., 2008; Akhondzadeh et al., 2009; Abbasi et al., 2012; Raison et al., 2013). Certain polymorphisms in genes associated with inflammation are associated with the risk for the development of mood disorders and treatment response (Bufalino et al., 2013; Michopoulos et al., 2015). Together, these findings implicate inflammation in a subset of persons with depression who likely exhibit unique variations in pathobiology and clinical presentation.

Fascinatingly, parallel evidence demonstrates that a lack of physical activity (PA) promotes the accumulation of visceral fat, adipose infiltration by proinflammatory immune cells, persistent low-grade inflammation (Ouchi et al., 2011), and, thereby, an increased risk for depression (Leonard, 2007). Conversely, adequate levels of persistent PA exert positive immunomodulatory (Hamer and Steptoe, 2007; Walsh et al., 2011) and antidepressant effects (Cooney et al., 2013; Schuch et al., 2016), even in persons who did not remit with conventional antidepressant treatment (Trivedi et al., 2011). Some reports suggest that PA outcomes compare favorably to antidepressant and cognitive behavioral therapy in mild to moderate depression (Mead et al., 2009). The therapeutic effect of long-term PA on depression likely includes the optimization of neurotransmitter level and function, hormone regulation, muscle-derived protein (e.g., peroxisome proliferator-activated receptor C coactivator-1α [*Pgc-1*α] and IL-6), and neurotrophic factors (Phillips, 2017a). Within contracting skeletal muscles, PA elicits intermittent elevations of IL-6 (Pedersen et al., 2001; Pedersen and Fischer, 2007), which then induces the synthesis of IL-10 and inhibits the release of TNF-α (Schindler et al., 1990; Apostolopoulos et al., 2014; Silverman and Deuster, 2014). Upon release into the local and systemic circulation, IL-10 promotes an anti-inflammatory milieu in the periphery. In the long-term, PA appears to lower levels of proinflammatory cytokines by altering visceral fat mass (de Lemos et al., 2012; Sell et al., 2012) and Toll-like receptors (TLRs) (Lambert et al., 1985; Francaux, 2009; Gleeson et al., 2011; Sell et al., 2012; Drummond et al., 2013), changes that may be particularly beneficial to persons with comorbid depression and metabolic disorders given that activation of TLRs contribute to the development of insulin resistance (Francaux, 2009; Liang et al., 2013). Consideration of its inexpensive low-risk profile and ease of implementation (Barbour et al., 2007) has increasingly led to the suggestion that PA can be deployed as a therapeutic strategy to reduce the degree of depressive symptoms (Cooney et al., 2013; Pemberton and Fuller Tyszkiewicz, 2016) in mild to moderate depression (Carek et al., 2011; Stanton and Reaburn, 2014; Kvam et al., 2016; Schuch et al., 2016) in all age groups (Abu-Omar et al., 2004; Motl et al., 2004).

Because it is important that clinicians and scientists understand the means by which PA can exert immunomodulatory effects in depression from both a self- and patient-education perspective, the aims of this review are to (1) underscore the link between inflammatory correlates and depression, (2) explicate immuno-neuroendocrine foundations, (3) elucidate evidence of monoaminergic and cytokine crosstalk in depressive pathobiology, (4) articulate the immunomodulatory mechanisms and pathways that confer the benefits of PA in depression, (5) examine protocols used to effectuate the benefits of PA in depression, and (6) highlight implications for clinicians and scientists. It is our contention that a deeper understanding of the mechanisms by which inflammation contributes to the pathobiology of depression will translate to novel and more effective treatments, particularly by identifying relevant patient populations that can benefit from immune-based therapies within the context of personalized medicine.

## Immuno-neuroendocrine foundations

**Figure 1:**
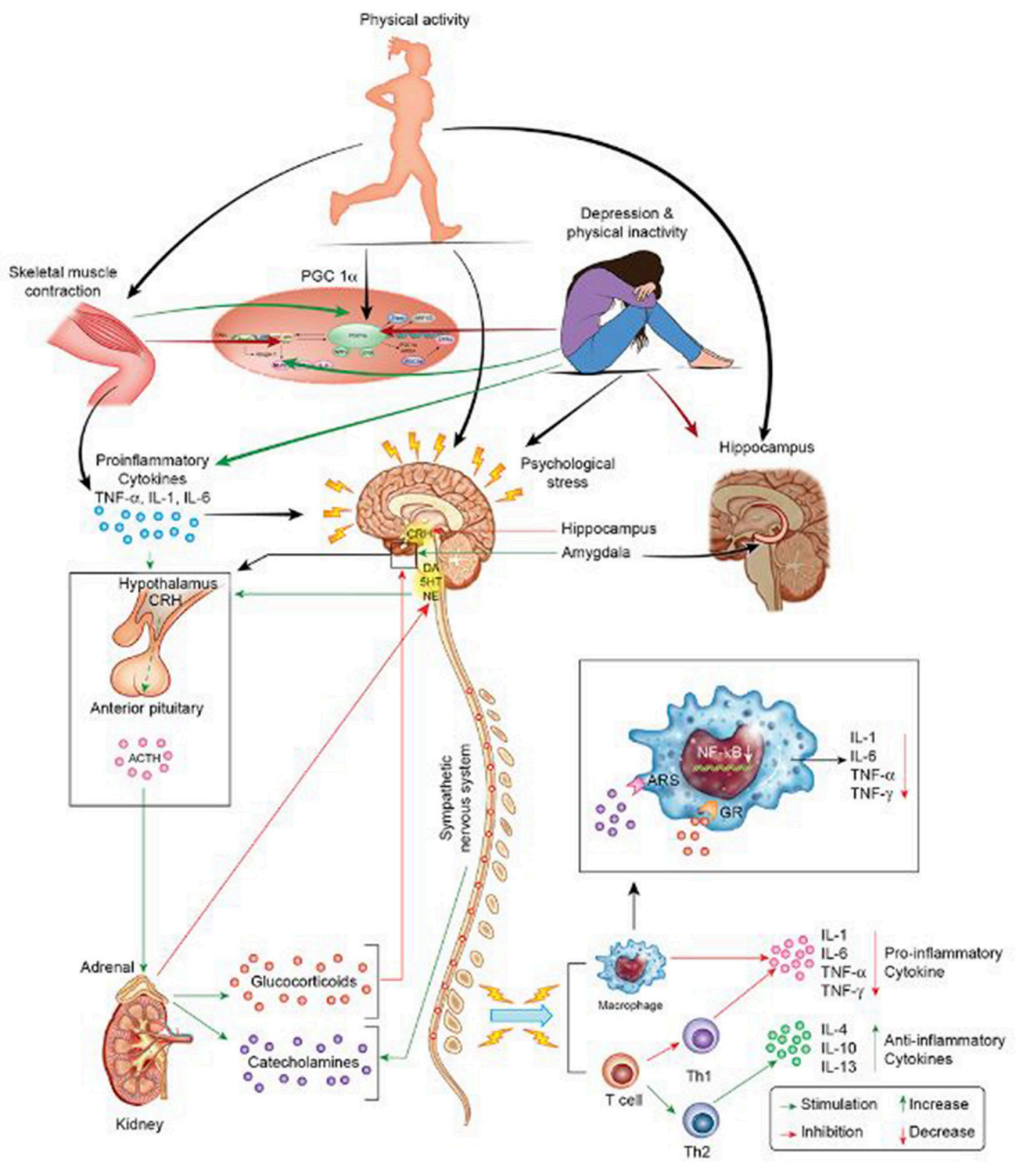
Stress, inflammation, and depression. The HPA and sympathetic nervous systems regulate the response to stressors, i.e., cytokines, psychological stress, and PA. The systemic response to stress is initiated via CRH secretion by the hypothalamus. CRH stimulates the pituitary to secrete ACTH into systemic circulation. In turn, ACTH secretion stimulates the adrenals to release catecholamines and glucocorticoids, factors that collectively induce pro- or anti-inflammatory cytokine release. Negative feedback mechanisms limit the process of inflammation in times of health. Conversely, persistent stress leads to dysregulation of the HPA with resultant endocrine disturbances in states of disease, e.g., depression. Stress-related disturbances in neuroendocrine hormones are problematic as they disrupt immune modulation and lead to a pro-inflammatory state. By acting as an intermittent stressor, PA exerts its' central and peripheral neuroprotective effects via several avenues. During PA, muscle contractions induce the release of myokines. These factors increase the expression of PGC-1α and decrease the expression of pro-inflammatory cytokines at the molecular level. Moreover, PA directly modulates neurotransmitter level and function (e.g., noradrenergic function), which is important promoting a pro- or anti-inflammatory milieu. Finally, PA increases hippocampal neurotrophic factor levels (e.g., BDNF) to promote hippocampal health and, thereby, promotes stress hormone regulation (e.g., cortisol regulation).

Within the context of homeostatic challenge, stressors initiate behavioral and immune responses so as to favor vigilance and protection against injury and immune challenge in lieu of explorative activities. The hypothalamic-pituitary-adrenal (HPA) axis and sympathetic nervous system coordinate these activities following activation by endogenous or exogenous stressors (Tsagarakis et al., 1989; Besedovsky and Rey, 2007). Specifically, exposure to psychological and physiological stressors activates the paraventricular nucleus of the hypothalamus. Activation of the paraventricular nucleus results in the release of corticotropin-releasing hormone (CRH), which then stimulates the release of adrenocorticotropic hormone (ACTH) from the pituitary. This, in turn, induces cortisol and catecholamine secretion from the adrenals (Figure 1). Initially, the HPA and sympathetic nervous system function to increase cortisol and catecholamine release during challenge to coordinate the fight-or-flight response. These stress hormones inhibit excess production of proinflammatory cytokines (e.g., IL-12, TNF-α, and interferon [IFN]-γ) in healthy individuals with optimal regulatory capacity, while simultaneously increasing production of anti-inflammatory cytokines (e.g., IL-10 and IL-4) (Elenkov and Chrousos, 1999). Concomitantly, cortisol exerts inhibitory effects upon the hypothalamus and pituitary (Crosby and Bains, 2012) through medial prefrontal cortex (mPFC) receptors (Hill et al., 2011a,b) and reduces stress-induced over excitability of the amygdala (Gray et al., 2015) in conditions of health. Temporal and sequential regulation of the immune response is paramount as failure in negative feedback effectuates persistent hypersecretion of proinflammatory cytokines, which can then induce central neuroinflammation (Leonard, 2001; Lacy and Stow, 2011) via active transport mechanisms at the circumventricular organs, binding to blood vessel receptors, or by retrograde transport by the vagus nerve (Maier and Watkins, 2003). By accessing the brain via the aforementioned mechanisms, systemic inflammation can activate resident microglia in the brain (McCusker and Kelley, 2013) following peripheral immune challenge (D'Mello et al., 2009) and promote depression in vulnerable individuals.

Undoubtedly, persistent systemic inflammation alters the function and expression of glucocorticoid receptors in the HPA axis, changes that impair negative feedback mechanisms (Karanth et al., 1997) at the level of the hypothalamus and anterior pituitary (Besedovsky and Rey, 2007). Chronic stress results in lower diurnal cortisol secretion as well as a blunted stress response (Peeters et al., 2003), which is problematic for immune modulation given the anti-inflammatory characteristics of catecholamines (e.g., noradrenaline (Bergmann et al., 1999) and cortisol (Cupps and Fauci, 1982). Whereas basal levels of noradrenaline promote an anti-inflammatory milieu (Bergmann et al., 1999), depletion of noradrenaline promotes a proinflammatory milieu, an effect that can be blocked by the beta adrenergic receptor agonist isoproterenol (Madrigal et al., 2005). Additionally, disruption of the catecholamine response is deleterious to the brain given that noradrenaline modulates the neuroprotective effects of astrocytes via trophic factor release (Junker et al., 2002). Much evidence clearly demonstrates that HPA dysregulation and prolonged inflammation contribute to depressive pathobiology (Pariante and Miller, 2001).

Thus, on the one hand the immune system defends against endogenous and exogenous stressors. On the other hand, it acts as a regulatory system that is in continual communication with the nervous and the endocrine systems via reciprocal communication mediated by cytokines, hormones, and neurotransmitters (Besedovsky and Rey, 2007). Imbalances among these mediators induce chronic disease conditions such as depression. Some persons with depression exhibit activated cell-mediated immunity with a T helper (Th)1-style response and elevated levels of IFN-γ (Maes et al., 1990; Maes, 2011), whereas others exhibit a distinct subtype wherein a Th2-style response predominates (Fornaro et al., 2013), possibly reflective of different stressor profiles, disease points, or genetic contributions.

At the molecular level, pro-inflammatory cytokine interactions with their cognate receptors initiate signaling events that promote a feedforward inflammatory process if left unchecked. I_k_B proteins typically sequester inactive transcription factors in the cytoplasm in unstimulated cells. Yet in states of low-grade inflammation, persistent receptor stimulation by cytokines and TLR agonists triggers intracellular signaling events that activate I_k_B kinase (IKK) activity and induce the dissociation of the I_k_B protein complex and, in turn, promote I_k_B degradation. The resultant release of nuclear factor κB (NF_k_B) dimers (e.g., p50/p60) permits their translocation to the nucleus and binding to cognate DNA sites that then regulates transcription of inflammatory genes and antioxidant defense (Baldwin, 1996).

Within the context of metabolic disorders, it has become increasingly evident that the presence of persistent pro-inflammatory cytokines plays a vital role in certain depressive phenotypes. The global prevalence of depression is approximately 5% in the general population; and yet, this figure approximates 20% or more in persons with obesity, diabetes, and coronary artery disease (Ferrari et al., 2013b), subsets of the population that may be particularly resistant to conventional antidepressant therapy (Raison et al., 2013; Felger et al., 2016; Haroon et al., 2016). The low-grade inflammation that results in these persons derives in part from macrophages and T-cells infiltration of adipocytes in white adipose tissue, liver, and skeletal muscle. This infiltration elicits a state of persistent secretion of proinflammatory cytokines, including TNF-α, IL-1, and IL-6 and a reduction in anti-inflammatory cytokines (e.g., adiponectin) (Hotamisligil, 2006; Kanda et al., 2006; Pedersen, 2009; Ouchi et al., 2011). The proinflammatory cytokines IL-1, IL-6, and TNF-α are thought to play a primal role in the neurotransmitter and neuroendocrine changes that occur in depression given their central role in sickness behavior. Undoubtedly, the presence of pro-inflammatory cytokines markedly affects neurotransmission within regulatory brain circuits related to emotions and induces hormonal changes commensurate with those observed following stress (Gadek-Michalska et al., 2013).

## Neurotransmitter and cytokine interactions in depression

Heretofore, we have established how depression is associated with peripheral and central inflammation and, thereby, how anti-inflammatory agents mitigate symptoms. Now we review evidence that inflammatory cytokines alter the level and function of key neurotransmitters that are relevant to depression neurobiology. We also explicate how cytokines activate the kynurenine pathway, lower tryptophan levels, and produce metabolites that modulate dopamine and glutamate function. We then shown how anti-inflammatory therapeutics (pharmacotherapy and PA) can optimize monoamine neurotransmitter level and function by modulating the synthesis, metabolism, and release of serotonin, noradrenaline, dopamine, and glutamate (Phillips, 2017a). Via these mechanisms, inflammation and therapeutics that mitigate depression may dramatically alter its pathobiology by directly impinging on the levels and function of key neurotransmitters that regulate depression circuit function and integrity and, ultimately, the affected individual's emotional and cognitive health.

### Serotonergic interactions

The majority of central serotonin-synthesizing neurons within the brain derive from the raphe nuclei, which are located near the midline of the brainstem (Das et al., 2014). The raphe sends more than 500,000 terminals to the cortical and limbic system (Cowen, 1991). This immense number of connections enable the raphe to modulate mood (Canli and Lesch, 2007), appetite (Blundell, 1984), arousal (Dubovsky, 1994), impulsivity (Dubovsky, 1994), aggression (Passamonti et al., 2012), and the sleep-wake cycle (Monti, 2011). Serotonin is synthesized from dietary tryptophan and translocated to the central nervous system, a rate-limiting step in serotonin synthesis. The fact that the serotonergic dorsal raphe is juxtaposed to the cerebral aqueduct suggests a vulnerability to inflammation. Indeed, evidence demonstrates that proinflammatory cytokines alter the functional status of the serotonergic system in the raphe and beyond in a manner similar to that seen in depression, providing a mechanistic explanation for the serotonergic abnormalities and associated symptoms seen in persons with depression.

Direct evidence that cytokines contribute to serotonergic dysfunction in depression pathobiology derives from data from electrophysiological, neurochemical, genetic, behavioral models, as well as from translational investigations. Intracellular recordings performed in a guinea-pig brain stem slice preparation demonstrated that IL-1β decreased spontaneous firing rates of serotonergic neurons by 50%, an effect that was reversible with washout (Manfridi et al., 2003). A parallel investigation in rodents showed that IL-1β inhibited the firing of dorsal raphe serotonergic neurons by enhancement of GABA-ergic inhibitory tone (Brambilla et al., 2007). These electrophysiological findings are congruent with the idea that IL-1 promotes non-rapid eye movement sleep by inhibiting the spontaneous firing of wake-active serotonergic neurons in the dorsal raphe nucleus (Brambilla et al., 2007). By corollary, the latter findings suggest that IL-1 alterations may contribute to alterations in arousal in depression, particularly to disturbances in stage III and IV sleep (Jones et al., 1987). Other work demonstrates that peripheral immune challenge alters the release and metabolism of central serotonin across brain regions (Dunn, 1992; Palazzolo and Quadri, 1992; Cho et al., 1999), changes that alter transporter activity. Serotonin transporters are responsible for transporting serotonin from the synaptic cleft to the presynaptic neurons to terminate signaling. As a high-affinity transporter, serotonin transporters maintain low extracellular serotonin levels in the synapse to prevent overstimulation of receptors and ensure responsiveness. Within this context, it has been determined that chronic exposure to proinflammatory cytokines alters serotonin transporter activity in a regionally specific manner and, thereby, modulates serotonin levels in the nerve terminal (Haase and Brown, 2015). Some preclinical evidence shows that stress-induced activation of the 5-HT2a serotonin receptor decreased hippocampal brain-derived neurotrophic factor (BDNF) levels (Vaidya et al., 1999).

Fortunately, several lines of work suggest that chronic PA modulates the serotonergic system to mitigate depressive symptoms in persons with inflammation. Translational studies demonstrate that plasma-free tryptophan is increased following PA (Davis et al., 1992; Melancon et al., 2012) by catecholamine-induced elevations in lipolysis and non-esterified fatty acids that displace albumin-bound tryptophan (Horowitz and Klein, 2000). Elevations in free tryptophan levels in the periphery increase tryptophan availability to the brain and, in turn, enhance serotonin synthesis (Chaouloff et al., 1986).

It seems logical that serotonin levels are more readily maintained with chronic PA given that it reduces proinflammatory markers (e.g., IFN-γ and TNF-α) and increases anti-inflammatory markers (e.g., IL-6 and IL-10) (Petersen and Pedersen, 1985; Smith et al., 1999; Panagiotakos et al., 2005; Kohut et al., 2006; Liu et al., 2013). For example, persons with depression exhibit a decrease in the pro-inflammatory cytokine TNF-α after submax exercise along with an increase in anti-inflammatory IL-4 (Hallberg et al., 2010). Within adipose tissue, PA limits proinflammatory cytokine secretion and inhibits macrophage infiltration phenotypic switching (from pro- to anti-inflammatory) of macrophages (Kawanishi et al., 2010). Within immune cells and skeletal muscles, PA decreases TLR4 and TLR2 expression (Gleeson, 1985; Lambert et al., 1985; Francaux, 2009), changes that decrease the inflammatory capacity of leukocytes and may alter whole-body chronic inflammation (Gleeson et al., 2006). Studies of human peripheral blood following PA demonstrate a reduction in circulating proinflammatory monocytes (Timmerman et al., 2008) and an increase in circulating regulatory T cells (Yeh et al., 2006).

Undoubtedly, the ability of PA to bias the immune system toward an anti-inflammatory state is significant for the serotonergic system because this state downregulates IDO activity and shifts the ratio of kynurenine metabolites toward neuroprotective kynurenic acid and away from neurotoxic quinolinic acid (Ito et al., 2003; Kiank et al., 2010). Moreover, the reduction of pro-inflammatory cytokines by PA reduces serotonin uptake by transporters to increase serotonin in the nerve terminal (Mössner et al., 2001). Finally, the ability of PA to optimize levels of tryptophan and serotonin exerts positive effects on BDNF and neurogenesis in the prefrontal cortex and hippocampus, underscoring the interrelationship between these two signaling systems (Mattson et al., 2004; Ernst et al., 2006; Esch and Stefano, 2010).

### Noradrenergic interactions

Noradrenergic neurons are a vital component of the central “stress circuitry” that induces “fight or flight” behavior, fear, and anger. Noradrenergic synthesizing neurons are primarily located in the locus coeruleus (LC), a brainstem structure within close proximity to the fourth ventricle (Phillips et al., 2016). The LC sends extensive projections to a number of brain regions including the thalamus, frontal and entorhinal cortices, basal lateral amygdala, and hippocampus (Loughlin et al., 1986). The LC's vast arborization and divergence of collaterals permit widespread synaptic and extrasynaptic release of neurotransmitters and neuropeptides throughout the central neuraxis onto neuronal and non-neuronal cells (Fornai et al., 2007, 2011), enabling the LC to exert a significant modulatory effect on behavior and HPA axis secretion. The latter occurs via dense noradrenergic projections from the LC to corticotropin-releasing hormones in the paraventricular nucleus of the hypothalamus, enabling psychological stress and immune challenges to activate the hypothalamus and trigger glucocorticoid release (Gadek-Michalska et al., 2013). Robust evidence demonstrates that proinflammatory cytokines alter the functional status of the noradrenergic system in the LC and beyond, providing a mechanistic explanation for the noradrenergic abnormalities and associated symptoms seen in persons with depression.

Direct evidence that cytokines can contribute to noradrenergic dysfunction derives from electrophysiological, neurochemical, genetic, behavioral models, and from translational studies. Microinjection of IL-1 into the LC region increased firing activity of LC neurons in the rat brain, an effect that was blocked by an IL-1 antagonist. Intraperitoneal injection of a low dose of lipopolysaccharide increased LC firing activity, an effect that lasted 3 weeks after injection (Borsody and Weiss, 2002). IL-2 and IFN-α administration altered LC electrical activity (De Sarro et al., 1990; Nisticò and De Sarro, 1991; Nisticò, 1993). The ability of inflammation to increase LC activity is significant because a common characteristic of effective antidepressants is their ability to decrease LC neuronal activity. Moreover, LC activity is highly correlated with arousal: higher rates of neuronal firing occur during the awake state, whereas complete LC neuronal inhibition occurs during rapid eye movement sleep (Hobson et al., 1974; Aston-Jones and Bloom, 1981), suggesting that alterations in firing patterns in depression may contribute to the sleep disturbances seen in depression. Juxtaposed with the electrophysiological studies are neurochemical investigations that showed that a common response to cytokine release involved an increased noradrenaline metabolism in multiple brain regions (Dunn et al., 1989), but with a preferential activation of the ventral component of the system, which derived from the nucleus tractus solitarius and LC (Dunn, 1988). Lipopolysaccharides IL-1, IL-2, and IFN-α potently activate the noradrenergic system across brain regions (De Sarro et al., 1990; Dunn, 1992; Smagin et al., 1996), a change that is not surprising because sustained stress increases noradrenergic requirements. Pharmacological manipulations that increase noradrenergic action and duration at the synapse (e.g., noradrenergic reuptake inhibitors) elevate mood and attention, mitigating the effects of stress-mediated noradrenergic depletion.

Outside the brain, noradrenaline can modulate autonomic sympathetic postganglionic fibers via primary and secondary lymphoid organs (bone marrow and thymus versus spleen and lymph nodes, respectively) during immune challenge. These actions are accomplished via direct activation of β_2_-adrenergic receptors that are present on Th1 cells, but not on Th 2 cells (Sanders et al., 1997). Purportedly, the anti-inflammatory effects of β_2_-adrenergic receptors activation stem from the inhibition of Th1 pro-inflammatory cytokines (e.g., IFN-γ, IL-12, TNF-α) or stimulation of Th2 anti-inflammatory cytokines (IL-10, IL-6, or TGF-β) (van der Poll et al., 1996; Elenkov et al., 2000). It has been shown that noradrenaline suppresses IL-12 production in a dose-dependent fashion and at physiological concentrations, whereas it dose-dependently increases the production of IL-10, effects that are blocked completely by propranolol, a β-adrenergic receptor antagonist (Elenkov et al., 1996). These findings suggest that immune balance is regulated via peripheral end-effectors of the stress system and that chronic dysregulation may bias the system toward a pro-inflammatory status (Elenkov et al., 1996).

Several lines of evidence suggest that PA modulates the noradrenergic system directly and indirectly to mitigate depressive symptoms in persons with inflammation. Within minutes, PA activates the sympathetic nervous system in an activity-dependent manner to modulate the secretion of the adrenal hormone adrenaline. Similarly, within minutes PA activates release of ACTH from the hypothalamus. These intermittent hormonal responses are vital for an anti-inflammatory milieu because intermittent elevations in adrenaline (Bergmann et al., 1999) and cortisol exert anti-inflammatory effects (Cupps and Fauci, 1982). Most studies of PA have reported a higher adrenaline response post exercise in endurance trained persons as compared to untrained controls (Zouhal et al., 2008). Other work has shown that running elevates cortisol levels in saliva and plasma in healthy persons (Duclos et al., 1998; Labsy et al., 2013) when performed at 60% of VO2max (maximum capacity of oxygen uptake) (Labsy et al., 2013). The intermittent nature of PA also contributes to a proportional increase in inactivation of the active steroid (cortisol) into the inert steroid (cortisone). The ability of PA to optimize catecholamine and cortisol levels is paramount in persons with comorbid depression and inflammation because these hormones modulate immune function and yet may be blunted as a consequence of chronic stress.

Within the brain, PA induces the neuronal adaptation that is requisite for mitigating stressful stimuli in varied ways across brain regions. Preclinical study demonstrates that 6 weeks of wheel running reduces LC firing following stress (Greenwood et al., 2003; Greenwood and Fleshner, 2008). Underlying these adaptive effects (Greenwood and Fleshner, 2008) is the upregulation of galanin in the LC, which induces a hyperpolarization of noradrenergic neurons and, thereby, inhibits excessive noradrenaline release (Seutin et al., 1989; Pieribone et al., 1995; Reiss et al., 2009; Murray et al., 2010) in some brain regions. The latter changes are essential for reducing noradrenaline levels in the amygdala to limit anxiety behavior (Sciolino and Holmes, 2012). Recapitulating these findings in humans, it has been shown that galanin increases in plasma after acute episodes of PA (Legakis et al., 2000). Conversely, long-term PA increases noradrenaline levels in the hippocampus to improve cognitive outcomes (Sarbadhikari and Saha, 2006), a finding that may have important implications for microglia and astrocytes in this brain region. The maintenance of basal levels of noradrenaline is important for inhibiting the release of the proinflammatory cytokines by microglia (Feinstein et al., 2002; Mori et al., 2002) and stimulating astrocytes to release trophic factors (e.g., BDNF) for neuroprotection (Junker et al., 2002). Prospective randomized controlled trials have demonstrated that hippocampal volumes increase following long-term aerobic PA (i.e., 1–2 years) (Erickson et al., 2011; Rosano et al., 2017).

### Dopaminergic interactions

The majority of dopaminergic neurons are found in the ventral tegmental area (VTA) of the midbrain, the substantia nigra pars compacta, and the arcuate nucleus of hypothalamus. The dopaminergic neurons of these areas project to different brain structures through the mesocortical (with neurons originating in VTA and transporting dopamine to the amygdala, hippocampus, septum, and prefrontal cortex), mesolimbic (with neurons originating in the VTA and transporting dopamine to the nucleus accumbens through the amygdala and hippocampus), and nigrostriatal pathways (with neurons originating in the substantia nigra and transporting dopamine to the hippocampus and dorsal striatum that is comprised of the caudate nucleus and putamen) (Prasad and Pasterkamp, 2009). The diverse origins and ramifications of these pathways explain the varied effects produced by dopaminergic activation (Cho et al., 2006). Whereas optimal signaling in the mesolimbic pathway induces feelings of enjoyment and reinforcement following exposure to pleasurable stimuli (e.g., food, sex, and drugs) and associated contexts (Maas et al., 1997), optimal signaling in the mesocortical is vital for concentration and working memory. In the nigrostriatal system, signaling modulates motoric (planning and execution) and cognitive responses. In contrast, decrements in dopaminergic neurotransmission can effectuate symptoms of impaired ability to experience pleasure (anhedonia), motivation, executive function, and motricity in persons with depression (Nestler and Carlezon, 2006; Tye et al., 2013), a cluster of symptoms that traditional SSRIs often fail to assuage (Dunlop and Nemeroff, 2007; Trivedi et al., 2008). Knowledge that proinflammatory cytokines alter the functional status of the dopaminergic system in a similar manner to that seen in depression (reduces ventral striatal activity to reward cues) suggests a mechanistic explanation for their co-occurrence of inflammation in a distinct subset of persons who are clinically depressed.

Direct evidence that cytokines induce dopaminergic dysfunction derives from data from neurochemical, behavioral, electrophysiological, genetic, and human clinical studies. For instance, it has been shown that both peripheral and central administration of inflammatory agents alter dopamine levels in the brain (Miller et al., 2009), particularly in the striatum (Kamata et al., 2000; Mauriño et al., 2010). Interestingly, some evidence suggests cytokine-specific alterations across brain regions: IL-1 and IL-2 administration increased dopamine turnover in the prefrontal cortex, whereas IL-6 increased turnover in the hippocampus and prefrontal cortex (Zalcman et al., 1994). The effects of cytokine challenge may also be concentration and time dependent. Low concentration of IL-2 administered to mesencephalic cell cultures increased dopamine release, whereas higher concentrations had no effect (Alonso et al., 1993). A parallel *in vivo* micro dialysis study showed that acute treatment of monkeys with IFN-α increased dopamine release in the striatum, whereas chronic treatment with IFN-α decreased dopamine release. Notably, the decreased dopamine that occurred in the striatum after chronic treatment was correlated with reduced effort-based sucrose consumption (Felger et al., 2013b), an effect that was mitigated by levodopa administration via reverse *in vivo* microdialysis, suggesting that inflammatory cytokines reduce the availability of dopamine precursors without affecting end-product synthesis or vesicular packaging or release (Felger et al., 2015). Other work demonstrated that immune challenge effectuated decreased intracranial self-stimulation of lateral hypothalamus (Borowski et al., 1998). One of the structures affected by intracranial self-stimulation is the medial forebrain bundle which contains ascending dopaminergic projections from the VTA to the nucleus accumbens (mesolimbic pathway) and passes through the lateral hypothalamus (You et al., 2001; Nestler and Carlezon, 2006). Under basal conditions, the dopaminergic neurons in the VTA area oscillate between low-frequency regular action potentials (tonic activity) and bursts of action potentials (phasic activity patterns) (Schultz, 2002). Transient increases in phasic firing are thought to occur with exposure to unexpected rewards or aversive stimuli, thereby encoding a “reward prediction error” and reinforcing certain behaviors (Tsai et al., 2009). Notably, inflammatory stimuli decrease rodent responding for rewarding electrical stimulation in the lateral hypothalamus (Anisman et al., 1998; Borowski et al., 1998), a change that likely reflects anhedonia secondary to a loss of reward function. Translating this work to humans, it has been shown that volunteers exposed to low-dose polysaccharide exhibited reduced ventral striatal activity to monetary reward cues, a change that correlated with increased depressive symptoms (Eisenberger et al., 2010). It appears that there is a rate of long burst-like spike activity that is requisite for VTA dopaminergic neurons to release sufficient levels of dopamine to promote feelings of reward (Yadid and Friedman, 2008), a phenomenon that may be deleteriously altered in depression and inflammation. Indeed, administration of desipramine to Flinders-sensitive line rats (an animal model of depression) increased the rate of long-bursting, high-spike activity in the VTA in a manner similar to that seen in controls (Yadid and Friedman, 2008). Additionally, integrated behavioral, pharmacological, optogenetic, and electrophysiological methods used by Tye et al. (2013) to assess freely moving rodents showed that inhibition or excitation of dopaminergic neurons in the VTA immediately and bi-directionally modulated (induced or relieved) depressive-like symptoms effectuated by chronic mild stress.

One of the implicated mechanisms by which inflammation can alter dopaminergic signaling is via modulation of tetrahydrobiopterin (BH4), a cofactor that is essential for the degradation of amino acid phenylalanine and biosynthesis of dopamine. Chronic immune challenge correlates with reduced dopamine synthesis (Neurauter et al., 2008). In fact, patients treated with IFN-α demonstrate reduced BH4 function in the brain (Felger et al., 2013a). By corollary, persons subjected to dietary depletion of dopamine (via a tryptophan-depleting beverage) exhibited blunted activation of the ventral striatum during reward anticipation activities (Bjork et al., 2014), recapitulating the effects of immune challenge (Eisenberger et al., 2010). Further attesting to the effects of inflammation and dopaminergic function is recent work in a subgroup of clinically depressed individuals that showed that infliximab (a highly selective TNF-α antagonist) effectuated a strong antidepressant effect (with greatest effects seen in area of motivation), but only in patients with elevated CRP at baseline (Raison et al., 2013).

Interestingly, most studies suggest that PA increases dopamine levels in several brain regions (Brown et al., 1979; de Castro and Duncan, 1985; Dishman, 1997; Meeusen et al., 1997; Soares et al., 1999). These effects putatively stem from the ability of PA to alter metabolism (de Castro and Duncan, 1985; Chaouloff et al., 1986) via modulation of calcium levels (Goffer et al., 2013; Morris and Berk, 2015) and calcium/calmodulin-dependent activation of tyrosine hydroxylase (Greenwood et al., 2011) and mitigate BH4 depletion by inhibiting iNOS induction (Kitagami et al., 2003). Greenwood et al. (2011) demonstrated that young adult male Fischer rats that participated in voluntary wheel—running for 6 weeks exhibited a conditioned place preference for the wheel as well as increased ΔFosB/FosB immunoreactivity in the nucleus accumbens, increased tyrosine hydroxylase mRNA levels in the VTA, and compensatory down-regulation of D2 receptor mRNA in the nucleus accumbens; these findings suggest that (1) long-term voluntary PA is rewarding and alters gene transcription in mesolimbic reward neurocircuitry (Greenwood et al., 2011), (2) post-exercise increases in serum Ca^2+^ may activate tyrosine hydroxylase enzyme and dopamine synthesis, and (3) PA may reverse inflammation-induced disruptions in dopaminergic transmission in the nucleus accumbens and ventral striatum in models of depression. Another study showed that wheel running increases tyrosine hydroxylase mRNA in the LC (Droste et al., 2006) and substantia nigra (Foley and Fleshner, 2008). Receptor-binding studies suggest that 9 weeks of voluntary PA induced hypersensitivity to dopamine release (Gilliam et al., 1984; MacRae et al., 1987). In line with the aforementioned, it has been suggested that voluntary wheel running alters behavior because the activity is intrinsically rewarding and affects neuroplasticity in the mesolimbic reward pathway (Greenwood et al., 2011). Thereby, PA could serve as a feed-forward mechanism and further increase PA, a phenomenon that would reduce inflammation and metabolic disease in the long- term (Waters et al., 2008).

### Glutamatergic interactions

Excitatory glutamatergic neurotransmission provides a basis for communication in the forebrain, cortex, hippocampus, caudate nuclei, thalamic nuclei, and cerebellar nuclei (Paul and Skolnick, 2003). Once released into the synaptic cleft, glutamate acts upon pre- and post-synaptic ionotropic N-methyl-d-aspartate (NMDA) glutamate receptors (NMDARs) in brain regions that modulate monoaminergic activity, emotionality, learning, and behavior (Ghasemi et al., 2014, 2017). NMDARs are tetrameric structures comprised of 7 subunits, including an obligatory GluN1 subunit along with various combinations of GluN2 and GluN3 subunits that differ according to anatomical distribution, developmental profile, and functional activity. Multiple binding sites exist on NMDARs, including those for glycine (D-serine), Mg^2+^, and other polyamines.

Some evidence suggests that the fronto-limbic glial alterations that occur in depression (Rajkowska and Miguel-Hidalgo, 2007) and comorbid inflammation are the result of an imbalance between the quinolinic acid and kynurenic acid arms of the pathway. Cytokine activation of the kynurenine pathway induces the breakdown of kynurenine into either quinolinic acid or kynurenic acid. The two end- products have diametrically opposing functions that can contribute to neurodegenerative or neuroprotective processes in the brain. Quinolinic acid is a neurotoxic endogenous NMDA receptor agonist, whereas kynurenic acid is a neuroprotective endogenous NMDA receptor antagonist. Within the brain, quinolinic acid is exclusively produced in microglial cells, intimating that microglial activation by proinflammatory cytokines may bias quinolinic acid production and facilitate NMDA agonism (Myint et al., 2007; McNally et al., 2008) along with astrocytic activation and apoptosis (Guillemin et al., 2005). The loss of astrocytes is particularly problematic because they uptake synaptic glutamate to prevent neuronal excitotoxicity, provide metabolic support to neurons via lactate production, produce neuroprotective mediators, and defend against oxidative stress. Quinolinic acid agonism of extrasynaptic NMDARs also inhibits the activity of cAMP response element binding (CREB) protein to block induction of BDNF gene expression (Hardingham et al., 2002). Interestingly, the NMDA antagonist ketamine decreased lipopolysaccharide-induced TNF-α production in astrocyte and microglial cultures (Shibakawa et al., 2005). In the hippocampus, ketamine down-regulated pro-inflammatory cytokines (Wang et al., 2015), an effect that might reduce depression-related hippocampal atrophy and preserve HPA axis feedback (Chen and Guillemin, 2009; Leonard and Maes, 2012). Memantine, another NMDA antagonist, has been shown to mitigate lipopolysaccharide-induced neuroinflammation and restore behaviorally- induced gene expression and spatial learning in the rat (Rosi et al., 2006). Together, these studies suggest that inflammation-induced astrocytic pathology may play an important role in depression via the production of pathogenic substances and loss of normal function. By corollary, strategies that normalize astrocytic function may improve neuronal health and decrease microglial activation.

Notably, evidence suggests that PA positively modulates the glutamatergic system in states of depression and inflammation. PA increases glutamate turnover and prevents excitotoxicity (Jia et al., 2009; Herbst and Holloway, 2016) by improving calcium regulation (Sutoo and Akiyama, 1996). PA also exerts a neuroprotective effect on the brain by modulating glial function. Recently it was shown that rodents exposed to long-term PA (5 days per week × 4 weeks) demonstrate increased BDNF synthesis and release in the dentate gyrus along with altered orientation and morphology of astrocytes (Fahimi et al., 2017). The latter findings suggest the antidepressant effects of aerobic PA may stem in part from (1) PA-induced changes in astrocytic projection length and density that enhance glutamate clearance from the synapse to mitigate glutamate excitotoxicity and (2) astrocytic production of neuroprotective mediators. Others have shown that PA induces an increase in astrocyte cell body area and number of contacts between astrocytic endfeet and blood vessels in the hippocampus, medial prefrontal cortex, and orbitofrontal cortex (Brockett et al., 2015). Since astrocytic endfeet express glucose transporters (Iadecola and Nedergaard, 2007), it seems plausible that PA-induced upregulation of astrocytic endfeet contact with blood vessels may serve as a means to respond to intense energy demand (van Hall et al., 2009), an adaptation that may be particularly important during inflammation and depression. Finally, it has been shown that PA reduced age-related microglial proliferation rate in aged mice 1.5-fold as well as the number of activated microglia 1.8-fold, suggesting that PA reduced inflammatory molecules that stimulate microglia proliferation (Kohman et al., 2012).

## At the nexus of antidepressant efficacy and PA: PGC-1α

The nuanced relationship between PA and immune function is complex and incompletely understood, particularly in depression. On the one hand, extreme PA results in inflammation and immunosuppression. On the other hand, moderate PA promotes an anti-inflammatory environment (Gleeson, 1985). At the nexus of PA and immune interactions is a regulator of adaptation: the muscle-derived protein peroxisome proliferator-activated receptor C coactivator-1α (PGC-1α). PA upregulates skeletal expression of PGC-1α (Irrcher et al., 2003; Russell et al., 2003), which is important because this factor controls pro-inflammatory gene expression in muscle partly via inhibition of the NF-κB pathway. The NF-κB pathway contributes to cytokine production and cell survival (Eisele et al., 2013). A reification of the aforementioned concept can be seen in persons with comorbid depression and Type-2 diabetes.

**Figure 2:**
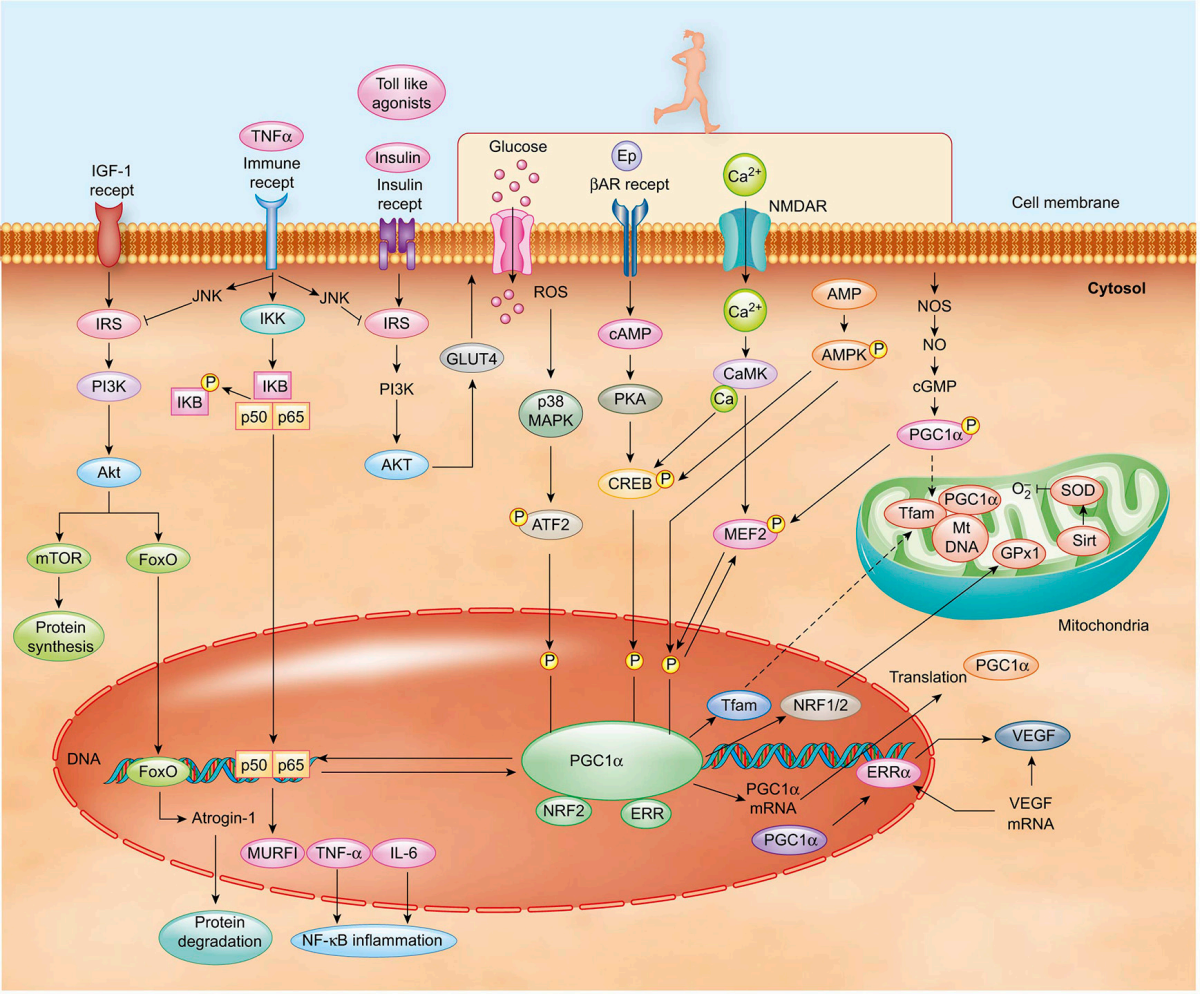
PA induces the upregulation of PGC-1α expression via multiple signaling pathways. Included among the pathway inputs are contributions from β-adrenergic receptor signaling, Ca^2+^, AMPK, ROS, and NO. Cytosolic PGC-1α protein translocates to the nucleus and mitochondria once activated. Various transcription factors can modulate metabolic processes, including MEF2, FoxO, ATF, and CREB. In turn, the factors are impinged upon by a multiplicity of signaling pathways. For instance, PA and cytokines activate p38 MAPK, which then induces the activation of MEF2 and ATF2. Insulin activates AKT, which then inhibits FoxO. PGC-1α and NF_k_B family p60 subunits reciprocally modulate one another to regulate inflammatory pathways.

Persons with Type-2 diabetes exhibit persistently elevated basal levels of inflammatory cytokines, which contribute to a state of insulin resistance (Hotamisligil, 2006). Low-grade inflammation putatively results when macrophages inundate white adipose tissue, liver, and skeletal muscle and elicit the persistent secretion of several types of “adipokines,” including the proinflammatory cytokines TNF-α, IL-1, IL-6, and monocyte chemoattractant protein-1 (MCP1) (Skurk et al., 2007). Interestingly, muscle tissue levels of TNF-α and IL-6 negatively correlate with PGC-1α levels in healthy and glucose-intolerant models (Handschin et al., 2007). Therefore, it seems plausible that the reciprocal regulation of PGC-1α and NF-κB is the molecular pivot in skeletal muscle that determines the balance between the trained anti-inflammatory environment endemic to conditions of health and the atrophic pro-inflammatory conditions endemic to states of disease (Figure 2). To better understand this pivot, a closer examination of IL-6 becomes warranted.

IL-6 is produced in skeletal muscle and adipose tissue, with adipose tissue contributing 10% to 35% of the body's basal circulating IL-6 level, a percentage that increases alongside rising body fat composition (Mohamed-Ali et al., 1997; Fried et al., 1998; Pedersen and Febbraio, 2012). Chronically elevated baseline IL-6 plasma levels are associated with obesity, insulin resistance, and Type-2 diabetes (Kern et al., 2001; Duncan et al., 2003; Dandona et al., 2004). Obesity-related elevations in IL-6 appear to help fuel the process of low-grade inflammation that accompanies obesity in a feedback response designed to offset energy excess. Bolstering the latter notion is evidence that PA increases IL-6 mRNA expression (Ostrowski et al., 1998; Starkie et al., 2001) in a manner that is contraction and duration dependent (Steensberg et al., 2000), suggesting that IL-6 signals the liver to increase glucose output to regulate blood glucose concentration during times of energy need (Steensberg et al., 2000). Other work shows that IL-6 can increase up to 100-fold with prolonged PA (Fischer, 2006), a trend that was attenuated with carbohydrate ingestion during PA (Nehlsen-Cannarella et al., 1985; Starkie et al., 2001) and pre-exercise glycogen depletion (50%) (Steensberg et al., 2001). Others showed that cytokines can induce white fat browning in peripheral tissue to promote energy expenditure (Lee et al., 2013; Petruzzelli et al., 2014). Furthermore, the induction of cytokine release by NFκB p65 in fat tissue induces energy expenditure in mice (Tang et al., 2010; Jiao et al., 2012). Notably, the chronic IL-6 and TNF-α secretion that results from obesity induces suppressor of cytokine signaling proteins (SOCS) 1 and 2. The net effect is a decrease in insulin-induced activation of insulin receptor substrate (IRS) with a reduction in the metabolic effects of insulin (Tanti et al., 2012) and failed skeletal muscle regeneration and atrophy (Coletti et al., 2005) via mechanisms that likely involve upregulation of TLRs (Lambert et al., 1985; Francaux, 2009; Gleeson et al., 2011; Drummond et al., 2013). With time, the hyperinsulinaemic response results in a decline in secretory capacity of β-cells that are responsible for insulin secretion. Conversely, administration of salicylate or blocking of IKK kinase reversed obesity and diet-induced insulin resistance (Gao et al., 2003; de Alvaro et al., 2004). Similarly, exercise improves insulin sensitivity and glucose uptake in muscle (Wojtaszewski et al., 2000, 2003; Sakamoto et al., 2004). Implicated mechanisms include increased phosphorylation of insulin receptor substrate (IRS) (Weigert et al., 2006).

Paradoxically, endurance-trained athletes exhibit increased levels of intramuscular triglycerides and yet are highly insulin sensitive (Goodpaster et al., 2001). Yet unlike sedentary individuals with comorbid depression and Type-2 diabetes, endurance-trained athletes appear to exhibit a higher mitochondrial density and mitochondrial enzyme capacity, which enhances oxidative phosphorylation and reduces the degree of insulin-sensitizing metabolic byproducts (Attie and Kendziorski, 2003; Mootha et al., 2003; Patti et al., 2003; Tarnopolsky et al., 2007). Moreover, the exercise-induced IL-6 profile in athletes differs from that with chronic inflammation. Whereas IL-6 is released from contracting muscle fibers following flux in Ca^2+^ and glycogen in exercising athletes (Pedersen, 2009), it appears to be primarily elicited from TLRs in persons with inflammation. Together, the aforementioned evidence suggests that an acute elevation in IL-6 exemplifies an attempt to mitigate energy crises during times of deprivation or excess, but that chronic inflammation promotes sickness behaviors, muscle wasting, and insulin resistance. Undoubtedly, the maintenance of metabolic equilibrium during inflammation involves PGC-1α, the master regulator of energy expenditure and mitochondrial biogenesis.

PGC-1α co-localizes to mitochondria-rich tissues, including skeletal muscle, liver, and brain. Transgenic studies of PGC-1α in rodents suggest the factor modulates local and systemic inflammation, including levels of TNF-α and IL-6 (Handschin and Spiegelman, 2008; Handschin, 2009; Arnold et al., 2011). The ability of PGC-1α to respond to changing metabolic needs during inflammation stems from its ability to selectively bind transcription factors, particularly peroxisome proliferator-activated receptor (PPAR)γ (Puigserver and Spiegelman, 2003), PPARα (Vega et al., 2000), estrogen-related α (ERRα) (Huss et al., 2002), forkhead box O (FoxO) (Puigserver et al., 2003), hepatocyte nuclear factor 4α (HNF4α) (Yoon et al., 2001), and nuclear respiratory factors (NRFs) (Wu et al., 1999). These coregulators affect biological responses that enable cells modulate mitochondrial biogenesis, cellular respiration rates, and energy substrate uptake and utilization—changes that are particularly important for contractile and metabolic adaptations in skeletal muscle (Puigserver and Spiegelman, 2003; Wende et al., 2007; Scarpulla, 2008). For example, PGC-1α coactivation of NRF-1,2 elicits the expression of nuclear-encoded mitochondrial proteins and mitochondrial transcription factor A (Tfam) to stimulate mitochondrial DNA replication and transcription (Kelly et al., 2003; Puigserver and Spiegelman, 2003; Lin et al., 2005). Cell culture studies of myoblasts show that overexpression PGC-1α effectuates an upregulation in respiratory subunit mRNAs, cytochrome c oxidase subunit 4 (COXIV) protein levels, and steady-state levels of mitochondrial DNA (Wu et al., 1999) in an adaptation to facilitate increased oxygen utilization. PGC-1α activity is regulated after PA via translational modifications that include phosphorylation (Jäger et al., 2007), deacetylation (Cantó et al., 2009), and sumoylation (Rytinki and Palvimo, 2008), changes that enhance expression of target genes and PGC-1α itself. Several pathways appear to contribute to these exercise-related modifications, including Ca^2+^/calmodulin, AMPK, p38/MAPK, and nitric oxide (NO) pathways.

PGC-1α activity is partially regulated by muscle-induced Ca^2+^ changes and their downstream signaling pathways. PA induces Ca^2+^ signaling via calmodulin-dependent protein kinase IV (CaMKIV) and calcineurin A, changes that activate myocyte enhancer factor (MEF) 2 (which is important for glucose transport) and impinge upon PGC-1α transcription (Handschin et al., 2003). Interestingly, transgenic mice overexpressing calcineurin A in skeletal muscle exhibit increased slow twitch myofibers, glucose transporter type 4 (GLUT4), mitochondrial enzymes, and PGC-1α (Naya et al., 2000; Ryder et al., 2003), suggesting that PGC-1α may have an insulin-sensitizing role. Corroborating the latter notion are studies showing a negative correlation between muscle PGC-1α levels and mitochondrial activity in insulin resistance and diabetes (Attie and Kendziorski, 2003; Mootha et al., 2003; Patti et al., 2003). In an alternate signaling path, CaMKIV activates cAMP response element (CRE) binding (CREB) protein to augment PGC-1α transcription in various tissues (Herzig et al., 2001; Wu et al., 2002; Handschin et al., 2003) and, in an autoregulatory manner, activates MEF2C and MEF2D (Michael et al., 2001; Lin et al., 2005). Another factor that upregulates PGC-1α during PA is p38MAPK via activation of transcription factor 2 (ATF2) (Cao et al., 2004). Also, aerobic PA-induced Ca^2+^ release upregulates PGC-1α activity and initiates its translocation to the nucleus, where it interacts with LRP130 to inhibit transcriptional activity of FoxO, which suppresses muscle protein degradation and atrophy (Vechetti-Junior et al., 2016). Via these mechanisms, aerobic PA and its induction of PGC-1α influences muscle fiber type composition, modulates GLUT4 gene expression (Michael et al., 2001), and promotes protein synthesis in muscle cells.

PA also generates reactive oxygen species (ROS), which induces inflammatory cytokine production in skeletal muscle (Ji, 2008), an effect that can be mitigated by the upregulation of mitochondrial ROS-detoxifying enzymes via PGC-1α (St-Pierre et al., 2003; Valle et al., 2005). Deficits in PGC-1α secondary to disuse may promote an inflammatory state that attenuates early benefits of exercise, particularly in those with comorbid depression and chronic inflammation. Yet restoration of PA effectuates a reduction in the ubiquitin-proteasome actions of atrogin-1 and Murf-1, proteins that are involved in atrophy under catabolic conditions (Dupont-Versteegden et al., 1985; Haddad et al., 1985; Okamoto et al., 2011; Suetta et al., 2012), via upregulation of PGC-1α, a metabolic change that promotes muscle recovery by inhibiting the FoxO pathway, possibly by involvement of LRP130 (Vechetti-Junior et al., 2016).

PA also induces changes in AMP-activated protein kinase (AMPK), an energy sensor that becomes active when the AMP/ATP ratio is high (Jørgensen et al., 2005; Pedersen and Febbraio, 2012). Activated AMPK enhances mitochondrial biogenesis and function, for which PGC-1α plays an essential role in activation (Jäger et al., 2007). The up-regulation of PGC-1α putatively occurs following direct phosphorylation by activated AMPK (Jäger et al., 2007). Then, activated PGC-1α may exert significant impact on mitochondrial signal transduction by up-regulating the expression of ERRα, nuclear respiratory factor (NRF)-1, and NRF-2 (Ye et al., 2016), which is important for antioxidant defense (Asghar et al., 2007). Other work suggests that PGC-1α is requisite for the upregulation of skeletal muscle VEGF expression, an effect that is AMPK- mediated (Leick et al., 2009). In addition to its importance in muscle physiology, the AMPK pathway may be particularly important for central neurons that possess small energy reserves (Ronnett and Aja, 2008) as suggested by concomitant AMPK activation in the rodent hippocampus and antidepressant-like effects following ketamine administration (Xu et al., 2013). Via these complex pathways, PGC-1α mediates many known beneficial effects of PA in skeletal muscle physiology and immune function.

Additionally linking PGC-1α with depression is recent groundbreaking preclinical work that demonstrated that exercise-induced augmentation of PGC-1α directly influenced mood by altering the kynurenine pathway via immune-dependent mechanisms (Agudelo et al., 2014). Initially this work proved onerous because PGC-1α is expressed in a variety of systems throughout the body making it difficult to disentangle whether the effects of PA originated from central or peripheral mechanisms. To tackle the problem, Agudelo and colleagues used mice that were genetically modified to produce excessive levels of PGC-1α in type-II skeletal muscle fibers and exposed them to chronic stress in an attempt to induce depressive-like symptoms (Agudelo et al., 2014). They found that mice overexpressing PGC-1α were far more resistant to depressive symptoms in comparison to mice with normal levels of PGC-1α. The researchers then attempted to induce depressive-like symptoms in mice that were genetically engineered to produce lower levels of PGC-1α in their skeletal muscles. This time, after a significant amount of stress, the low PGC-1α mice appeared to “lose hope,” as evidenced by their decreased survival efforts during forced swimming (an indicator of depression), behaviors that were inflammation-dependent (Agudelo et al., 2014; Phillips and Salehi, 2016). Importantly, PGC-1α overexpression effectuated an increased production of kynurenine aminotransferase (KAT), an enzyme that converts kynurenine into kynurenic acid, a substance that cannot pass from the blood to the brain. The conversion of kynurenine into kynurenic acid has tremendous translational potential given that high levels of kynurenine are found in persons with mental illness and rodents administered kynurenine display depressive behavior. Fascinatingly, recent human studies show that aerobic PA increases skeletal muscle KAT levels and, thereby, shifts kynurenine metabolism in the periphery toward kynurenic acid (Schlittler et al., 2016). Altogether, these results suggest that PA induces the release of “hope molecules” from the skeletal muscles of rodents to influence mood disorder symptoms.

To date, much of the aforementioned work has not been extended to large-scale patient populations with comorbid depression and inflammation. Nevertheless, the work provides a strong theoretical basis for the idea that PA can modulate PGC-1α, increase mitochondrial density, alter muscle fiber type, mitigate inflammation, and reduce depressive symptoms, particularly in persons with comorbid depression and diabetes. Undoubtedly, the ability of PA to optimize insulin control would exert significant peripheral and central effects. Acute aerobic PA significantly increases muscle glucose uptake via insulin-dependent mechanisms for 1 h after cessation, and increased glucose uptake persists 12–48 h following prolonged activity via insulin-independent mechanisms (Magkos et al., 2008). Also, improvements in insulin signaling may persist for 24 h when the intensity is increased to near-maximal effort intermittently during trainings of shorter duration (20 min) (Manders et al., 2010; Gillen et al., 2012), with some evidence suggesting that those with the highest baseline insulin resistance yield the greatest effects early in disease progression (Dubé et al., 2012). High-intensity interval training robustly enhances skeletal muscle oxidative capacity and insulin sensitivity in adults with Type-2 diabetes (Cochran et al., 2014; Jelleyman et al., 2015). Similarly, resistance training enhances insulin action (Bacchi et al., 2013), and some evidence suggests that a combination of endurance and resistance exercise effectuates greater improvements (Sigal et al., 2007). Improved insulin sensitivity is paramount as insulin signaling regulates mitochondrial function, energy homeostasis, circuit structure and function (via transmitter receptor trafficking), and plasticity (via alterations in synapse density) (Chiu et al., 2008), effects that may be particularly important in the aging hippocampus (Zhao et al., 2008; De Felice et al., 2009) given its importance for HPA regulation.

So, the question arises as to whether there is currently enough evidence to support the deployment of PA to positively influence depressive symptoms in clinical populations. To answer this important question, Cooney and colleagues conducted a meta-analysis of randomized trials that were published up to March 2013 in which exercise (defined according to American College of Sports Medicine criteria) was compared to standard treatment, no treatment or a placebo treatment, pharmacological treatment, psychological treatment, or other active treatment in adults (aged 18 and over) with depression (Cooney et al., 2013). Thirty-nine studies with a total of 2,326 participants were included in the review. The authors reported that aerobic exercise produced effects comparable to treatment by either antidepressants or psychotherapy. Another meta-analytic study by Silveira and colleagues demonstrated that aerobic PA moderately reduced the signs of depression, with populations over 60 years of age and those with mild depression deriving the greatest response (Silveira et al., 2013). Notwithstanding, there is currently little evidence to indicate which modality of PA is optimal (aerobic, strengthening, flexibility, or combinations). Stanton and Reaburn tried to determine optimal parameters for using PA to treat depression (e.g., frequency, intensity, duration, and type of exercise). All five randomized controlled studies meeting inclusion criteria were aerobic in nature (walking on treadmill or outdoors, cycling on a stationary bike, or training on an elliptical machine) (Stanton and Reaburn, 2014). Positive evidence was found that aerobic PA of moderate intensity, undertaken 3 times weekly, was effective in treating depression, with the ultimate recommendation for duration being a minimum of 9 weeks (Stanton and Reaburn, 2014).

Given evidence that it may be more difficult for persons with comorbid depression and inflammation to benefit from conventional antidepressants, it seems likely that associating pharmacological and nonpharmacological interventions that reduce inflammation may enhance treatment response in persons with comorbid depression and inflammation. Bolstering this notion is evidence that inflammatory cytokines may cancel mechanisms requisite for antidepressant efficacy by increasing monoamine transporter activity, reducing monoamine precursors, reducing enzyme cofactors necessary for monoamine synthesis, activating NF-κB, and reducing glutamate transporters. Fornaro et al. (2013) reported that non-responders to duloxetine exhibited increased levels of proinflammatory cytokine levels in comparison to early-responders. Yoshimura et al. (2009) showed that antidepressant efficacy was contingent upon the restoration of pro- and anti-inflammatory balance and lowering of baseline IL-6 levels (Yoshimura et al., 2009). *Post-hoc* analysis of clinical trial results by Raison et al. (2013) demonstrated that persons with treatment-resistant depression and high baseline CRP (>5 mg/L) exhibited a higher rate of treatment response (62 vs. 33%) when administered infliximab as compared to a placebo-treated group. Conversely, persons with low CRP (<5 mg/L) levels who were administered placebo experienced a greater reduction in depressive symptoms in comparison to those administered infliximab, a finding that argues against administration of anti-inflammatory agents in cases of depression without apparent inflammation (Raison et al., 2013).

So the question arises as to what can be expected when combining antidepressants with PA: an enhanced effect, a lower antidepressant dose, a higher rate of responders, a decreased rate of relapse, or a reduction in the delay of action? To answer this question, Carneiro et al. (2015) administered pharmacotherapy plus 16 weeks of supervised structured aerobic exercise training program to women with clinical depression in a randomized clinical trial, finding that aerobic exercise was an effective adjuvant to pharmacological therapy (Carneiro et al., 2015). Helgadóttir et al. (2017) assessed outcomes for four interventions: treatment as usual, light intensity exercise, moderate intensity exercise, and vigorous exercise; while all groups experienced decrements in depressive symptoms, persons in light exercise group reported greater symptom relief at 12-month follow-up (Helgadóttir et al., 2017). Siqueira et al. (2016) reported that a 4-week (4x/week) add-on aerobic exercise program significantly decreased the need to render higher doses of sertraline as compared to sertraline monotherapy (Siqueira et al., 2016). Kerling et al. (2015) demonstrated that treatment response was more frequent in persons assigned to an add-on exercise group in comparison to treatment as usual (Kerling et al., 2015). Babyak et al. (2000) assessed the effects of a 4-month course of aerobic exercise, sertraline therapy, or a combination of exercise and sertraline in persons with depression, finding that remitted persons in the exercise group exhibited significantly lower relapse rates than subjects in the medication group (Babyak et al., 2000). Preclinical work suggests that both voluntary PA and antidepressant therapies induce changes in neuroplasticity substrates in a similar time course (Russo-Neustadt et al., 1999), although it remains to be determined whether PA-induced reductions in inflammation could alter the time course in those with comorbid depression and high basal levels of inflammation.

The precise activity parameters that need to be deployed to mitigate depression and comorbid inflammation need to be determined in future work. Some translational work suggests that moderate PA may be an optimal intensity of PA for the promotion of mental health by decreasing TNF-α (Paolucci et al., 2018). Clearly, PA prescriptions are needed that take into account the basal levels of inflammation and response to stress (neuroendocrine and immune status) during intervention. The end goal would be to deploy various forms of PA to intermittently stimulate the immune response so that the levels of pro-inflammatory mediators and stress hormones are optimized. Doing so may require different modalities, depending upon personal factors.

## Conclusions and future directions

Undoubtedly, psychiatric illness is defined by a constellation of different symptoms that can be influenced by multiple neural processes and circuits. The heterogeneity of presentations complicates the precise targeting of dysfunction and, by corollary, therapeutics that target those impairments. This conundrum is patently apparent in the management of depression, wherein a growing body of work suggests that a specific subtype of depression with comorbid chronic inflammation exists. Further research that aims to characterize the relationship between inflammation and depression is warranted as it may yield novel treatments for this subgroup that has been shown to be resistant to conventional antidepressant pharmacotherapy. Biomarker identification efforts will be enhanced by methods that triangulate protein and genetic analysis with neuroimaging and behavioral analyses. Ultimately, these studies should be used to identify the immune, endocrine, and neurotransmitter responses in depressive subtypes so that optimal treatments, both pharmacological and nonpharmacological, can be identified and tailored to select patient populations. Clearly, large-scale, multiple-site clinical investigations that study the relationship between PA and depression are needed. Longitudinal studies will be required to evaluate the short- and long-term benefits of combination therapies. The effects of PGC-1α on phenotypic traits—such as adiposity, lean mass, and fasting glucose—and the way that they could be modulated by genetic background (ethnicity) are not precisely understood. Studies that disentangle the relationship between PA, cognitive engagement, diet, social factors, and stress are desperately needed to determine the independent and additive protective effects that each factor exerts (Phillips et al., 2014, 2015; Phillips, 2017b,c), particularly how these factors affect cognitive and emotional function at the synaptic and circuit level (Das et al., 2015). Finally, strategies for overcoming the core symptoms of depression and comorbid health problems are needed so that PA prescriptions can be personalized and adherence maximized. Personalized prescriptions are particularly germane to the topic of depression as the condition is associated with increased morbidity and mortality (Kiecolt-Glaser and Glaser, 2002), and activation of the inflammatory response in persons with depression may engender different treatment responses to various activity regimens. These studies are vital to work at the frontiers of neuroscience that seeks to enable novel application of PA to health and disease and provide a personalized approach to intervention.

## Author contributions

CP developed the concept for the study. CP and AF were responsible for manuscript writing and editing.

### Conflict of interest statement

AF reports a consulting relationship with Silverberry Genomix, but acknowledges the company did not have a role in this paper. The remaining author declares that the research was conducted in the absence of any commercial or financial relationships that could be construed as a potential conflict of interest.

## Abbreviations

ACTH: adrenocorticotropic hormone
AMPA: α-amino-3-hydroxy-5-methyl-4-isoxazolepropionic acid
BH4: tetrahydrobiopterin
BDNF: brain-derived neurotrophic factor
CRH: corticotropin-releasing hormone
CREB: cAMP response element-binding protein
CRP: c-reactive protein
ERRα: estrogen-related α
FoxO: forkhead box O
GLUT4: glucose transporter type 4
HPA: hypothalamic–pituitary–adrenal
IDO, indolamine 2: 3-dioxygenase
IFN: interferon
IL: interleukin
IRS: insulin receptor substrate
KAT: kynurenine aminotransferase
KMO: kynurenine 3-monooxygenase
LC: locus coeruleus
MAPK: mitogen-activated protein kinase
MEF: myocyte enhancer factor
MHPG: 3-methoxy-4-hydroxypheny-glycol
NFkB: nuclear factor κB
NRF: nuclear respiratory factor
NMDA: N-methyl-D-aspartate
NO: nitric oxide
SOCS: suppressor of cytokine signaling proteins
PA: physical activity
Pgc-1α: peroxisome proliferator-activated receptor C coactivator-1α
SSRI: selective serotonin reuptake inhibitor
Th: T helper
TNF: tumor necrosis factor
TDO, tryptophan 2: 3-dioxygenase
TLRs: Toll-like receptors
VTA: ventral tegmental area.
